# Cell Infectivity in Relation to Bovine Leukemia Virus gp51 and p24 in Bovine Milk Exosomes

**DOI:** 10.1371/journal.pone.0077359

**Published:** 2013-10-17

**Authors:** Tetsuya Yamada, Hiroaki Shigemura, Naotaka Ishiguro, Yasuo Inoshima

**Affiliations:** Department of Veterinary Medicine, Gifu University, Gifu, Japan; Northeast Agricultural University, China

## Abstract

Exosomes are small membranous microvesicles (40–100 nm in diameter) and are extracellularly released from a wide variety of cells. Exosomes contain microRNA, mRNA, and cellular proteins, which are delivered into recipient cells via these exosomes, and play a role in intercellular communication. In bovine leukemia virus (BLV) infection of cattle, although it is thought to be a minor route of infection, BLV can be transmitted to calves via milk. Here, we investigated the association between exosomes and BLV in bovine milk. BLV structural proteins, gp51 (Env) and p24 (Gag), were detected in bovine milk exosomes from BLV-infected cattle by Western blot analysis. In cells inoculated with these milk exosomes, BLV DNA was not detected during three serial passages by nested PCR. Purification of exosomes from persistently BLV-infected cells was achieved by immuno-magnetic separation using an antibody against exosomes coupled to magnetic beads. Consistently, BLV gp51 and p24 proteins were detected in purified exosomes. Moreover, reverse transcriptase activity was observed in purified exosomes, meaning that exosomes also contain viral enzyme. However, BLV DNA was not detected in serially passaged cells after inoculation of purified exosomes, indicating that exosomes carrying BLV proteins appeared to be not infectious. These results suggest that BLV proteins are released with milk exosomes and could be transferred into recipient cells of calves via milk exosomes as an alternative route not requiring virus infection. Moreover it is also possible that bovine milk exosomes play a role in clearance of BLV proteins from infected cells.

## Introduction

Exosomes, which are small membranous microvesicles (40–100 nm in diameter), originate in endocytic compartments and are extracellularly released from a wide variety of mammalian cells [1]. In humans, exosomes are present in various physiological fluids, including plasma [2], [3], ascites [4], urine, amniotic fluid [5], [6], saliva, breast milk [3], [7], and bronchoalveolar lavage fluid [8]. Exosomes contain microRNA (miRNA), mRNA, and membrane and intracellular proteins [9]. Therefore, it has been suggested that exosomes play a role in intercellular (cell-to-cell) communication, such as activation/suppression of immune and cellular function, through either direct interaction of exosomal surface antigens with target cell receptors, or via the transfer of RNAs and proteins from fused exosomes into target cells [10].

During the past decade, it has been reported that exosomes released from virus-infected cells contain viral nucleic acids and proteins in some cases; this has been observed in both RNA and DNA virus infections in humans with human immunodeficiency virus (HIV) [11]–[14], hepatitis C virus [15], [16], herpes simplex virus [17], [18], and Epstein-Barr virus [19], [20]. These exosomes are considered to be involved with viral infection, pathogenesis and host defense systems [21], [22].

Bovine leukemia virus (BLV) belongs to the Genus *Deltaretrovirus* in the family *Retroviridae*. BLV is the causative agent of enzootic bovine leukosis, and BLV infection occurs worldwide [23]. Symptoms in cattle include lymphocytosis, lymphadenopathy, neuropathy, and progressive emaciation [23]. In BLV infection, cattle are frequently asymptomatic, and in many cases infected animals remain virus carriers for life without clinical symptoms. Approximately 30% of infected cattle develop persistent lymphocytosis [24]. BLV is mainly transmitted horizontally by direct exposure to biological fluid contaminated with BLV-infected lymphocytes, such as inappropriate re-use of injection needles and gloves for rectal examination [25], [26]. It is also known that infectious BLV particles and/or BLV-infected somatic cells are present in bovine milk. Although it is thought to be a minor route of infection, BLV can be transmitted to calves via milk [27]–[29]. However, details of the association between bovine milk exosomes and BLV pathogenesis are unknown.

In this study, both milk exosomes from BLV-infected cattle and exosomes from BLV-infected culture cells were characterized, and BLV transmission via exosomes was also investigated *in vitro*. We showed that milk exosomes from BLV-infected cattle contain BLV structural proteins, gp51 (Env) and p24 (Gag), but these exosomes seemed to be non-infectious to cells, or at least have low infectivity. Consistently, exosomes purified from BLV-infected cells by immuno-magnetic separation also contained BLV structural proteins and enzyme but infectivity to cells was not observed. These findings suggest that BLV proteins are released into milk exosomes, and could be transferred into recipient cells of calves via milk exosomes as an alternative route not requiring virus infection. Moreover it is also possible that bovine milk exosomes play a role in clearance of BLV proteins from infected cells.

## Materials and Methods

### Milk samples and isolation of exosomes

**Figure 1:**
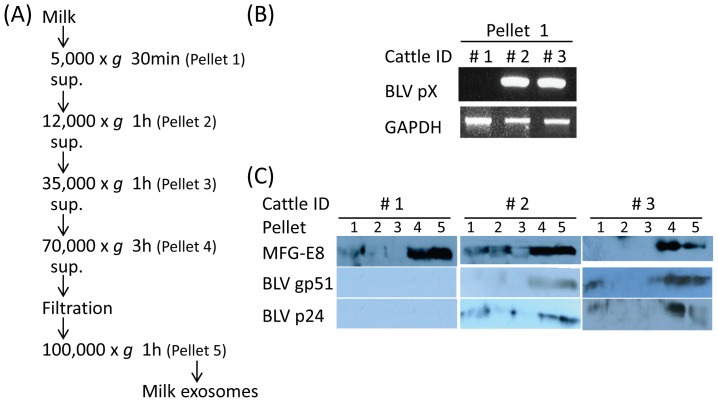
Exosome isolation from bovine milk. (A) Isolation procedure. At each step, the pellet (Pellet 1 to 5) was collected for detection of exosomes, BLV DNA and proteins. Supernatant was collected at each step and sequentially centrifuged for purification of milk exosomes. (B) Detection of BLV DNA in milk. BLV pX gene was detected by PCR in somatic cells in the milk (Pellet 1) of BLV-infected cattle #2 and #3, but not of healthy cow #1. Bovine GAPDH gene was used as an internal control for PCR targeting bovine DNA. (C) Detection of exosomes and BLV proteins in pellet at each step. Larger amounts of exosomes were detected in pellets 4 and 5 by WB analysis using anti-MFG-E8 antibody (an exosome marker). Similar to exosomes, larger amounts of BLV proteins gp51 (Env) and p24 (Gag) were detected in pellets 4 and 5 from BLV-infected cattle #2 and #3, but not from healthy cow #1.

This study did not include animal experiments for which approval was necessary, as judged by the Gifu University Animal Care and Use Committee. Milk from healthy (Cow ID #1) and BLV-infected cattle (#2 and #3) was purchased from farmers after routine milking and stored at −30°C until use. Exosomes from bovine milk (milk exosomes) were isolated by ultracentrifugation and sucrose-density gradient (SDG) centrifugation as described previously [30]. Bovine milk samples were centrifuged at 5,000× *g* for 30 min at 4°C in a T11A31 rotor (Hitachi Koki, Tokyo, Japan) using a Himac CF16RX centrifuge (Hitachi Koki) to remove milk fat globules (MFGs), as well as somatic cells and cell debris. From the cell pellet at this step, DNA was extracted and used in a polymerase chain reaction (PCR) to detect BLV DNA as described below. Defatted milk samples were then subjected to three successive centrifugation steps at 12,000× *g* for 1 h, 35,000× *g* for 1 h, and finally at 70,000× *g* for 3 h at 4°C in a P42A rotor using a Himac CP60E ultracentrifuge (Hitachi Koki) to remove residual MFGs, casein, and other debris (Figure 1A). The supernatant was filtered sequentially through 10.0-, 0.45-, and finally 0.22-µm filters (Millipore, Cork, Ireland). Filtered supernatant was ultracentrifuged at 100,000× *g* for 1 h at 4°C and the resulting pellet of milk exosomes was taken for Western blot (WB) analysis (Figure 1A). For further purification, milk exosomes were suspended in 1 ml of phosphate-buffered saline (PBS), layered on a linear SDG (5–40%, w/v) solution (9 ml), and ultracentrifuged at 200,000× *g* for 18 h at 4°C in a P40ST rotor (Hitachi Koki). Then, 0.9 ml of each gradient fraction was collected from the top of tube and numbered from 1 to 10. Each of the SDG fractions was diluted in 10 times the volume of PBS and ultracentrifuged again at 100,000× *g* for 1 h at 4°C. The pellet was gently suspended in a small volume of PBS and used for WB analysis and inoculation of cells.

### Cell cultures and isolation of exosomes from medium

Primary fetal lamb lung (FLL) cells [31] and fetal bovine muscle (FBM) cells [32] were cultured in Dulbecco's modified Eagle's medium (Wako, Osaka, Japan) with 10% fetal bovine serum (FBS), 100 µg/ml of streptomycin, and 100 U/ml of penicillin. Persistently BLV-infected fetal lamb kidney (FLK-BLV) cells [33] were cultured in Eagle's minimum essential medium (Wako) containing 10% XerumFree FBS replacement (TNCBIO, Eindhoven, Netherlands) instead of 10% FBS to exclude exosomes present in FBS. Exosomes from the culture medium of FLK-BLV cells were isolated by centrifugation at 3,000× *g*, filtration through a 0.22-µm filter, and ultracentrifugation at 100,000× *g*. The pellet of FLK-BLV exosomes was suspended in 1 ml of PBS, and taken for WB analysis, and further purification by immuno-magnetic separation as described below.

### WB analysis

Purified protein from each sample was used for WB analysis. Proteins separated on sodium dodecyl sulfate polyacrylamide gels were transferred to an Immobilon-P PVDF membrane (Millipore). The membrane was blocked with 5% non-fat dried milk in PBS containing 0.1% Tween20 (PBST) for 1 h at room temperature. The membrane was then incubated with primary antibodies for exosome markers; anti-bovine MFG-epidermal growth factor 8 (MFG-E8) (1∶100) [34] and anti-Flotillin-1 (1∶1000) (610820, BD Transduction Laboratories, Lexington, KY, USA), diluted in 1% non-fat dry milk in PBST for 1 h. Monoclonal antibodies specific to a D-D′ epitope on BLV gp51 and a linear epitope on BLV p24 (1∶1000), (BLV2 and BLV3, VMRD, Pullman, WA, USA) were also used for detection of BLV proteins. Goat anti-mouse IgG antibody conjugated with horseradish peroxidase (1∶1000), (NA9310V, GE Healthcare, Buckinghamshire, UK) was used as a secondary antibody, diluted in 1% non-fat dry milk in PBST. The bound immune complexes were detected using Amersham ECL Western Blotting Detection Reagents (GE Healthcare) and X-ray film.

### Immuno-magnetic separation of exosomes

FLK-BLV exosomes were further purified from the suspension by immuno-magnetic separation using an antibody against surface antigen of exosomes and immuno-magnetic beads. Dynabeads M-280 coated with sheep anti-mouse IgG (11201, Invitrogen Dynal AS, Oslo, Norway) were incubated with antibody against an exosome marker CD9 (LT86A, Veterinary Microbiology and Pathology, Washington State University, Pullman, WA, USA) or isotype control mouse IgG (X0943, Dako Cytomation, DK, Glostrup, Denmark) with slow rotation mixing for 1 h at 4°C. Anti-CD9 antibody combined with Dynabeads (anti-CD9/Dynabeads complex) and isotype IgG combined with Dynabeads (isotype IgG/Dynabeads complex) were washed with PBS. Normal healthy mouse serum (1∶1000) was added to the suspension of FLK-BLV exosomes for blocking and then the exosomes were incubated with either of these complexes for 1 h at room temperature with rotation mixing. The resulting exosomes/CD9/Dynabeads complexes were isolated using a magnet, washed with PBS, and used for WB analysis, inoculation of cells, observation by electron microscopy, and reverse transcriptase (RT) assay. After immuno-magnetic separation, the remaining unbound solution was ultracentrifuged at 100,000× *g* and the resulting pellet was used for WB analysis and inoculation of cells. Isotype IgG/Dynabeads complexes after incubation with the suspension of FLK-BLV exosomes were also isolated using a magnet and used for WB analysis and RT assay.

### Inoculation of cells with milk and FLK-BLV exosomes

Since FLL cells are known to support BLV replication efficiently [33], FLL as well as FBM cells were used for inoculation of exosomes. FLL and FBM cells in 6-well plates were incubated with milk exosomes from BLV-infected cattle for 1 h at 37°C. Exosomes/CD9/Dynabeads complexes obtained from the FLK-BLV exosome suspension by immuno-magnetic separation, as well as unbound solution, were also incubated with FLL and FBM cells. The cells were then washed twice with PBS, cultured, and were passaged 3 times at 4 day intervals. Cells were collected and DNA was extracted to detect BLV DNA by PCR. Before these experiments, the suitability of this assay system for assessing cell infectivity was tested and confirmed using FLL cells, by inoculation with the supernatant of FLK-BLV cells or the supernatant of FLL cells as a control, respectively (Figure S1).

### PCR

DNA was extracted from somatic cells in milk (Figure 1A, Pellet 1) using the DNeasy Blood and Tissue Kit (Qiagen, Hilden, Germany), and subjected to PCR using primers for BLV pX [35] and bovine glycelaldehyde-3-phosphate dehydrogenase (GAPDH) as a control [36] (Table S1). PCR was carried out in a 20 µl total reaction volume containing Go Taq Green Master Mix (Promega, Madison, WI, USA), 10 pmole of forward and reverse primers and 1 µl of extracted DNA samples. The amplification reaction was performed in a GeneAmp PCR system 9700 (Applied Biosystems, Foster City, CA, USA) as follows: initial denaturation for 2 min at 95°C, 35 cycles of denaturation for 30 s at 94°C, annealing for 30 s at 62°C, extension for 30 s at 72°C and a final extension at 72°C for 7 min. PCR products of BLV pX PCR were used for nested PCR. DNA from cells incubated with milk or FLK-BLV exosomes was also used for PCR.

### Electron microscopy

Magnetically separated exosomes/CD9/Dynabeads complexes were embedded in 20 µl of 3% low-melting point agarose and prefixed with 2% glutaraldehyde in 0.1 M phosphate buffer (pH 7.4) at 4°C. Samples were then cut into small pieces. The specimen was post-fixed with 2% osmium tetroxide in 0.1 M phosphate buffer (pH 7.4) for 1.5 h. These were dehydrated in a graded series of ethanol, finally with propylene oxide, and embedded in Quetol 812 epoxy resin (Nissin EM, Tokyo, Japan) for transmission electron microscopy. Ultra-thin sections (90–100 nm) were cut using a Ultracut-UCT (LEICA, Nussloch, Germany) with a diamond knife, and stained with 2% uranyl acetate in distilled water for 15 min, followed by a lead staining solution [37] for 5 min. Sections were examined with an H-7600 electron microscope (Hitachi, Tokyo, Japan) at 100 kV.

### RT assay

Viral RT activity in purified exosomes by immuno-magnetic separation was estimated by an improved RT assay using SYBR Green-based real-time PCR, which is known as product-enhanced RT (PERT) assay, as described previously [38], [39], with some modifications. Purified exosomes by immuno-magnetic separation were suspended in 50 µl of water and 5 µl of the suspension were mixed with 5 µl of 2× concentrated virus lysis buffer (0.25% Triton X-100, 50 mM KCl, 100 mM TrisHCl, pH 7.4, and 40% glycerol) containing 0.8 U/µl RiboLock RNase Inhibitor (EO0381, Thermo Fisher Scientific, Vilnius, Lithuania), and incubated for 10 min at room temperature. After dilution with 90 µl of water, the lysates were centrifuged at 4,000× *g* for 2 min at 4°C in a TMA-29 rotor (Tomy Seiko, Tokyo, Japan) using an MX-301 centrifuge (Tomy Seiko) and 90 µl of supernatant were recovered. For PERT assay, 9.6 µl of the lysates were mixed with 10.4 µl of reaction mix containing 10 µl of 2× Fast SYBR Green Master Mix (Applied Biosystems), 0.1 µl RNA from bacteriophage MS2 (0.4 µg/µl, Roche Diagnostics, Mannheim, Germany), 0.1 µl MS2 forward primer (100 µM), 0.1 µl MS2 reverse primer (100 µM), and 0.1 µl diluted RiboLock RNase Inhibitor (4 U/µl). Serially diluted moloney murine leukemia virus (M-MLV) RT (M5301, Promega, Madison, WI, USA) was used for construction of standard curve of RT activity in PERT assay.

### Statistical analysis

The data were analyzed for statistical significance by parametric non-repeated measures ANOVA and the post hoc test using SNK test. The data were collected from three independent experiments.

## Results

### Isolation of milk exosomes from BLV-infected cattle

**Figure 2:**
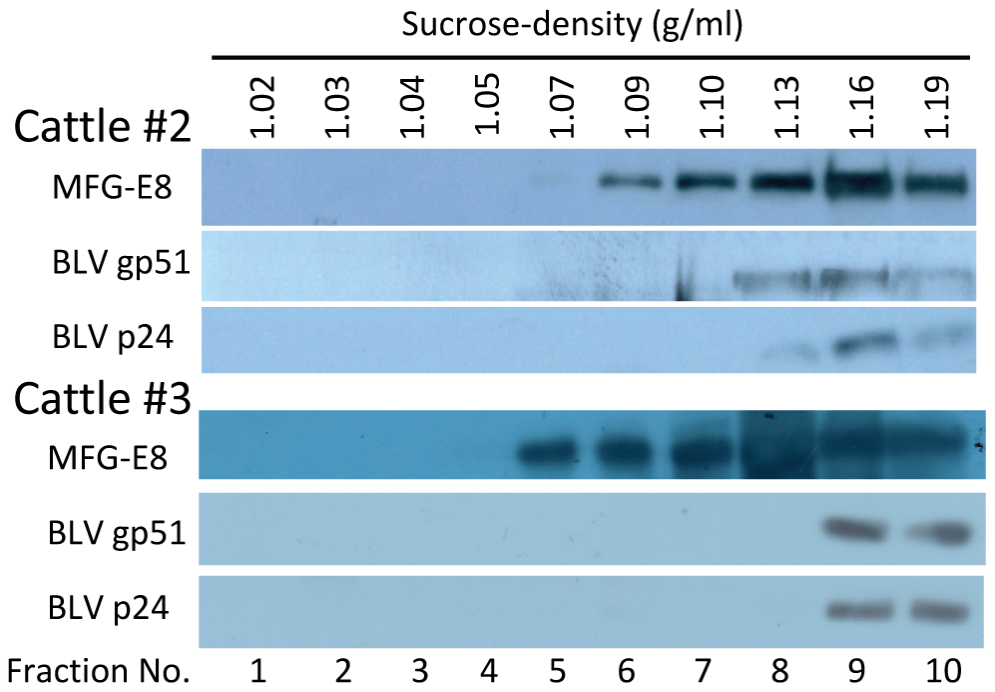
Detection of exosomes and BLV proteins in each fraction by SDG centrifugation. Pellet 5, from BLV-infected cattle #2 and #3 in Figure 1A was diluted in PBS, layered on the top of a linear SDG (5–40% w/v) solution and ultracentrifuged. Sequential fractions from SDG were collected from the top of the tube (numbered from 1 to 10). BLV proteins gp51 (Env) and p24 (Gag) were present at 1.13 to 1.19 g/ml (fraction nos. 8 to 10) in #2 and at 1.16 to 1.19 g/ml (fraction nos. 9 to 10) in #3, respectively, consistent with the fraction containing a greater number of exosomes (MFG-E8).

Although it is known that BLV-infected somatic cells are present in bovine milk [27]–[29], Kuckleburg *et al*. [28] showed that BLV DNA in milk was detected in only 65% (52/80) BLV-seropositive cattle. Moreover, Michishita *et al.* [40] reported that, even if BLV DNA was detected in blood, BLV DNA in milk was detected in only 35% (14/40) of cattle where BLV was detected in blood. Therefore, initially to confirm the presence of BLV DNA in milk, and so to allow the selection of milk samples containing BLV, PCR was carried out. BLV DNA was detected in somatic cells (Pellet 1) in milk from BLV-infected cattle #2 and #3 but not from healthy cow #1 (Figure 1B). Then, we tried to separate both the BLV proteins in somatic cells (Pellet 1), and free BLV particles, from milk by sequential centrifugation. Pellets at each step (Pellets 1 to 5) of the isolation procedure for milk exosomes (Figure 1A) were subjected to WB analysis. Large amounts of milk proteins such as casein, along with cell debris, were precipitated in pellets 2 and 3 as described previously [30] and these could not be dissolved efficiently in sample buffer for WB analysis. A larger amount of exosomes, which were identified by an exosome marker MFG-E8, were detected in pellets 4 and 5, indicating the necessity of sequential centrifugation steps for exosome isolation (Figure 1C). Similar to the exosomes, BLV proteins gp51 and p24 were detected in pellets 4 and 5 (Figure 1C). At this point, by WB analysis, we could not conclude that free BLV particles were excluded from the isolated milk exosomes. Thus, to further purify exosomes, pellet 5 from both BLV-infected cattle #2 and #3 was suspended in PBS, layered on SDG solution, and ultracentrifuged. Recovered sequential fractions from SDG were subjected for WB analysis. BLV proteins gp51 and p24 were present at 1.13 to 1.19 g/ml (fractions 8 to 10) from cow #2, and at 1.16 to 1.19 g/ml (fraction nos. 9 to 10) from cow #3, respectively; these fractions also contained a greater number of exosomes (MFG-E8) (Figure 2). These results demonstrated that purified milk exosomes contain BLV proteins. However, we could not completely exclude the possibility that free BLV particles were present in the exosome fractions, because it is reported that BLV particle density is 1.148-1.164 g/ml [41]. The difference of fractions containing BLV proteins between cattle #2 and #3 is probably due to the individual differences in milk, including age, milking period involving physical and chemical properties of milk, and viral load in milk. Therefore, to further purify exosomes and to exclude influences by unknown proteins or factors due to individual differences in milk, FLK-BLV exosomes were used for further experiments instead of milk exosomes.

### Exosome separation from culture medium of FLK-BLV cells

**Figure 3:**
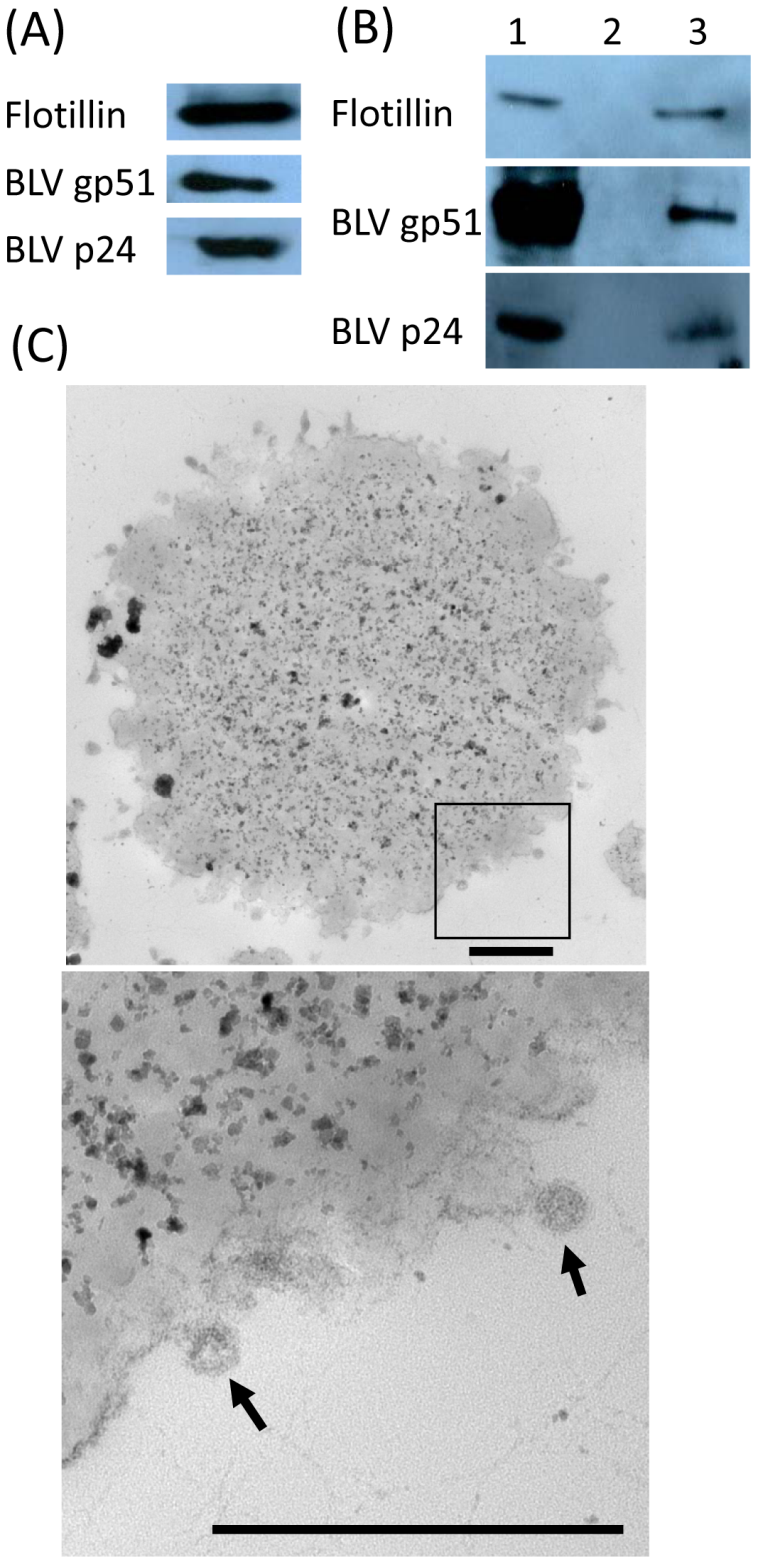
Exosome purification from culture medium of FLK-BLV cells using the anti-CD9/Dynabeads complex and magnetic separation. (A) WB analysis of FLK-BLV exosomes. Exosomes (Flotillin), BLV gp51 (Env) and p24 (Gag) proteins were detected in pellets obtained by ultracentrifugation at 100,000× *g* from the culture medium of FLK-BLV cells. (B) Immuno-magnetic separation of FLK-BLV exosomes. Lane 1, unbound solution after immuno-magnetic separation; lane 2, isotype IgG/Dynabeads complexes separated using a magnet; lane 3, exosomes/CD9/Dynabeads complexes separated using a magnet. To exclude the possibility of the presence of free BLV, similar amounts of exosomes (Flotillin) were present in both inocula (lanes 1 and 3). Larger amounts of BLV proteins in the unbound solution (lane 1) might indicate that this solution may still have contained both BLV proteins with exosomes, and free BLV particles. (C) Electron micrographs of exosomes/CD9/Dynabeads complexes obtained by immuno-magnetic separation. Upper panel: an electron micrograph of anti-CD9/Dynabeads complexes at low magnification. Bottom panel: enlargement of the square in upper panel. Exosomes captured by anti-CD9/Dynabeads complexes were observed around the surface of Dynabeads (2.8 µm in diameter). Arrows indicate exosomes. Scale bar shows 500 nm.

BLV gp51 and p24 proteins were detected in the FLK-BLV exosomes by antibodies against BLV proteins, gp51 and p24 (Figure 3A). Since it was known that FLK-BLV cells released infectious virus particles into the culture medium [33], to exclude the possibility of the existence of free BLV particle in the isolated FLK-BLV exosomes, immuno-magnetic separation using the anti-CD9 (surface marker of exosomes) antibody and magnetic beads was carried out. Exosome (CD9), BLV gp51 and p24 proteins were detected in separated exosomes/CD9/Dynabeads complexes, and also in remaining unbound solution, but not in separated isotype IgG/Dynabeads complexes (Figure 3B). The amount of exosomes in unbound solution was equivalent to that found in separated exosomes/CD9/Dynabeads complexes. On the other hand, the amount of BLV proteins in unbound solution was greater than that of BLV proteins in exosomes/CD9/Dynabeads complexes, indicating that exosomes were successfully separated from free BLV particles by anti-CD9/Dynabeads. These results were confirmed by electron microscopic observation, where exosomes captured by the anti-CD9/Dynabeads were observed around the Dynabeads (Figure 3C).

### RT activity in exosomes

**Figure 4:**
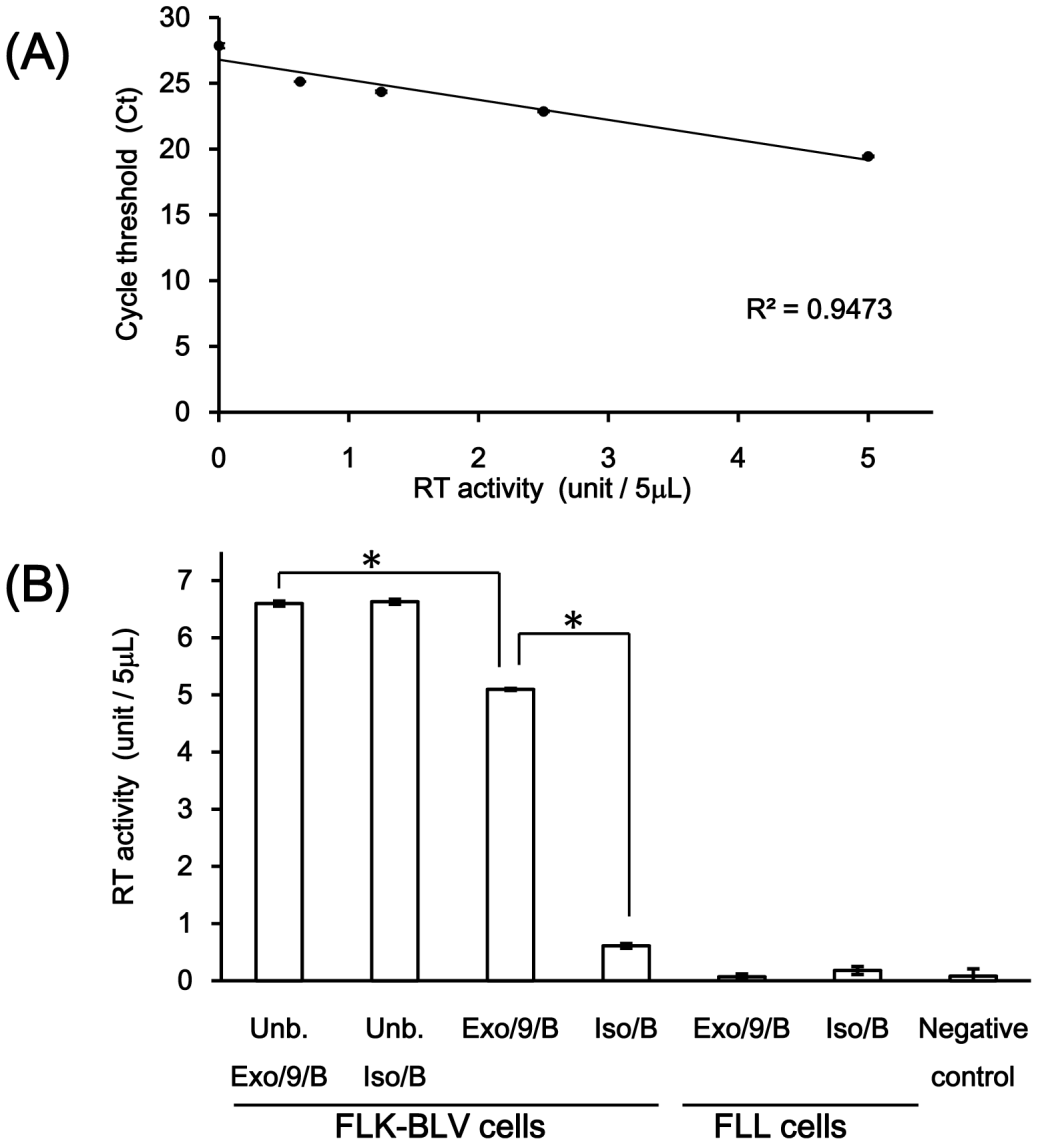
Reverse transcriptase activity in exosomes. (A) Standard curve of RT activity constructed by serially diluted M-MLV RT (5, 2.5, 1.25, 0.625 and 0 units/5 µl). (B) Detection of RT activity in purified exosomes by immuno-magnetic separation. Significantly higher activity in purified exosomes/CD9/Dynabeads complexes than isotype IgG/Dynabeads complexes was observed by parametric non-repeated measures ANOVA and SNK test. *, *P*<0.01. Unb. Exo/9/B; unbound solution of exosomes/CD9/Dynabeads complexes. Unb. Iso/B; unbound solution from the mixture of exosomes and isotype IgG/Dynabeads complex. Exo/9/B; exosomes/CD9/Dynabeads complexes. Iso/B; magnetically separated pellet from the mixture of exosomes and isotype IgG/Dynabeads complex. Negative control; water.

Viral RT activity was detected in purified exosomes/CD9/Dynabeads complexes as well as both unbound solution from exosomes/CD9/Dynabeads and isotype IgG/Dynabeads complexes (Figure 4). Viral RT activity was not detected in pellet from the mixture of exosomes and isotype IgG/Dynabeads complexes. No viral RT activity was detected in purified exosomes/CD9/Dynabeads and isotype IgG/Dynabeads complexes from FLL exosomes (Figure 4).

### Inoculation of cells with milk and FLK-BLV exosomes

Milk exosomes isolated from SDG fractions 7 to 9 were mixed and used to inoculate FLL and FBM cells. BLV infection was not detected at any passage of FLL and FBM cells by nested PCR (data not shown).

Exosomes/CD9/Dynabeads complexes from FLK-BLV cells, and the unbound solution after immuno-magnetic separation were also used to inoculate FLL and FBM cells. BLV DNA was not detected at any passage of FLL and FBM cells inoculated with exosomes/CD9/Dynabeads complexes, by nested PCR (Table 1). On the other hand, BLV DNA was detected at the first passage of FLL cells inoculated with unbound solution (Table 1). These results seemed to confirm that unbound solution still contained BLV, as described above.

**Table 1:** Infectivity of FLK-BLV exosomes to cells.

Cell^a^	Inoculum^b^	Passage number of cells^c^		
		1	2	3
FLL	Exo/9/B	−	−	−
	Unbound sol.	+	−	−
FBM	Exo/9/B	−	−	−
	Unbound sol.	−	−	−

a FLL; fetal lamb lung cells; FBM; fetal bovine muscle cells.

b Exo/9/B; exosomes/CD9/Dynabeads complexes obtained by immno-magnetic separation. Unbound sol.; unbound solution after immuno-magnetic separation.

c −; nested PCR negative, +; nested PCR positive.

## Discussion

In the present study, we confirmed for the first time the presence of BLV structural proteins, gp51 (Env) and p24 (Gag), in bovine milk exosomes from BLV-infected cattle, and also BLV enzyme, reverse transcriptase, in exosomes from BLV-infected cells. Previous studies showed that HIV Gag and Nef proteins were detected in exosomes from HIV-infected cells [11]–[14]. HIV Gag was sorted to endosome-like domains on the plasma membrane and secreted into exosomes from the cells. These exosomes contained prominent electron-dense cores of oligomerized HIV Gag [11], [13], suggesting the possibility that HIV can utilize the exosome biogenesis pathway for virion biogenesis and exosome uptake pathway as an alternative, Env-independent pathway for infecting neighboring cells, in a manner termed the “Trojan exosomes” hypothesis [42], [43]. Similar to HIV, BLV Gag proteins may be oligomerized in milk exosomes, released with exosomes, and delivered into recipient cells via these “Trojan exosomes”. In addition, bovine milk exosomes could be taken up by cells much more easily by phagocytosis because of MFG-E8 expression. Bovine milk exosomes express MFG-E8 on the surface, used as an exosome marker in this study. It is known that MFG-E8 mediates interactions between aminophospholipids on apoptotic cells or cell debris, such as phosphatidylserine, which acts as an “eat-me signal” to phagocytes, and α_v_β_3_ or α_v_β_5_ integrin on phagocytes [44], and then apoptotic cells and cell debris are engulfed by phagocytosis. Previously, proteome analysis identified 2107 proteins in bovine milk exosomes [45]. Moreover, bovine milk exosomes contained mRNAs of major milk proteins, such as caseins, β-lactoglobulin, and MFG-E8, and mammary gland- and immune-related miRNAs, and these RNAs were transferred into recipient cells [46]. In recent human studies, it has been reported that breast milk exosomes contain RNAs, miRNAs, and proteins, and deliver these RNAs and proteins into recipient cells, thus inducing gene expression and immune activation [3], [7], [47], [48]. These results suggest that RNAs and proteins, including BLV proteins, are released with milk exosomes and BLV proteins could be transferred into recipient cells of calves via milk exosomes as an alternative route not necessitating virus infection. Although the roles of BLV structural proteins and enzyme associated with exosomes were not elucidated in this study, it is possible that exosomes carrying BLV proteins could be involved with not only an alternative life cycle of BLV, but also in immunological responses against BLV in cattle like HIV in humans.

Additionally, a recent amyloid study using amyloid-β precursor protein (APP)-overexpressing transgenic mice has shown that, although there was no difference in the number of exosomes secreted from brain compared with wild-type mice, a larger amount of APP and the APP-processing products was observed in brain exosomes from the transgenic mice [49]. Interestingly, exosomes secreted from brain were enriched with APP and the processing products compared with brain homogenates in both transgenic and wild-type mice, suggesting a releasing system of neurotoxic APP and the products from the brain into the brain extracellular space [49]. These results suggest a probable biological significance that bovine milk exosomes may have a role in clearance of BLV proteins from infected cells.

Here, the infectivity of exosomes to cells was not observed *in vitro*. We used an efficient method for isolating morphologically native and highly purified bovine milk exosomes by ultracentrifugation with SDG centrifugation [30] and immuno-magnetic separation, which resulted in a small recovery of exosomes. It has been reported that exosomal purity, morphology, recovery, and RNA size and yield from exosomes differed between different isolation methods [30], [50], [51]. Previously, exosome-mediated infectious transfer of HIV [52], and prions 53,54 have been demonstrated. These results suggest that we still cannot ignore completely the possibility of BLV infection via milk exosomes and further detailed studies of BLV infection associated with milk exosomes are required both *in vitro* and *in vivo*.

## Supporting Information

Table S1 **Primers used in this study.**
(DOC)

Figure S1 **Infectivity assay, using FLL cells inoculated with the supernatant (sup.) of FLK-BLV cells.** BLV DNA was detected in passaged FLL cells after inoculation of supernatant of FLK-BLV cells but not in cells inoculated with supernatant of FLL cells as a control by nested PCR targeting the pX gene. Detection of the GAPDH gene in DNA from FLL cells was used as an internal control for the PCR reaction.
(TIF)

## References

[1] Théry C, Zitvogel L, Amigorena S (2002) Exosomes: composition, biogenesis and function. Nat Rev Immunol 2: 569–579. 12154376 10.1038/nri855

[2] Caby MP, Lankar D, Vincendeau-Scherrer C, Raposo G, Bonnerot C (2005) Exosomal-like vesicles are present in human blood plasma. Int Immunol 17: 879–887. 15908444 10.1093/intimm/dxh267

[3] Lässer C, Alikhani VS, Ekström K, Eldh M, Paredes PT, et al (2011) Human saliva, plasma and breast milk exosomes contain RNA: uptake by macrophages. J Transl Med 9: 9. 21235781 10.1186/1479-5876-9-9 PMC3033821

[4] Andre F, Schartz NEC, Movassagh M, Flament C, Pautier P, et al (2002) Malignant effusions and immunogenic tumour-derived exosomes. Lancet 360: 295–305. 12147373 10.1016/S0140-6736(02)09552-1

[5] Keller S, Rupp C, Stoeck A, Runz S, Fogel M, et al (2007) CD24 is a marker of exosomes secreted into urine and amniotic fluid. Kidney Int 72: 1095–1102. 17700640 10.1038/sj.ki.5002486

[6] Pisitkun T, Shen RF, Knepper MA (2004) Identification and proteomic profiling of exosomes in human urine. Proc Natl Acad Sci USA 101: 13368–13373. 15326289 10.1073/pnas.0403453101 PMC516573

[7] Admyre C, Johansson SM, Qazi KR, Filén JJ, Lahesmaa R, et al (2007) Exosomes with immune modulatory features are present in human breast milk. J Immunol 179: 1969–1978. 17641064 10.4049/jimmunol.179.3.1969

[8] Admyre C, Grunewald J, Thyberg J, Gripenbäck S, Tornling G, et al (2003) Exosomes with major histocompatibility complex class ΙΙ and co-stimulatory molecules are present in human BAL fluid. Eur Respir J 22: 578–583. 14582906 10.1183/09031936.03.00041703

[9] Valadi H, Ekström K, Bossios A, Sjöstrand M, Lee JJ, et al (2007) Exosome-mediated transfer of mRNAs and microRNAs is a novel mechanism of genetic exchange between cells. Nat Cell Biol 9: 654–659. 17486113 10.1038/ncb1596

[10] Pap E, Pállinger É, Pásztói M, Falus A (2009) Highlights of a new type of intercellular communication: microvesicle-based information transfer. Inflamm Res 58: 1–8. 19132498 10.1007/s00011-008-8210-7

[11] Booth AM, Fang Y, Fallon JK, Yang JM, Hildreth JEK, et al (2006) Exosomes and HIV Gag bud from endosome-like domains of the T cell plasma membrane. J Cell Biol 172: 923–935. 16533950 10.1083/jcb.200508014 PMC2063735

[12] Campbell TD, Khan M, Huang MB, Bond VC, Powell MD (2008) HIV-1 Nef protein is secreted into vesicles that can fuse with target cells and virions. Ethn Dis 18: S2–14–19. PMC3418053 18646314

[13] Fang Y, Wu N, Gan X, Yan W, Morrell JC, et al (2007) Higher-order oligomerization targets plasma membrane proteins and HIV Gag to exosomes. PLoS Biol 5: e158. 17550307 10.1371/journal.pbio.0050158 PMC1885833

[14] Lenassi M, Cagney G, Liao M, Vaupotič T, Bartholomeeusen K, et al (2010) HIV Nef is secreted in exosomes and triggers apoptosis in bystander CD4^+^ cells. Traffic 11: 110–122. 19912576 10.1111/j.1600-0854.2009.01006.x PMC2796297

[15] Masciopinto F, Giovani C, Campagnoli S, Galli-Stampino L, Colombatto P, et al (2004) Association of hepatitis C virus envelope proteins with exosomes. Eur J Immunol 34: 2834–2842. 15368299 10.1002/eji.200424887

[16] Tamai K, Shiina M, Tanaka N, Nakano T, Yamamoto A, et al (2012) Regulation of hepatitis C virus secretion by the Hrs-dependent exosomal pathway. Virology 422: 377–385. 22138215 10.1016/j.virol.2011.11.009

[17] McLauchlan J, Rixon FJ (1992) Characterization of enveloped tegument structures (L particles) produced by alphaherpesviruses: integrity of the tegument does not depend on the presence of capsid or envelope. J Gen Virol 73: 269–276. 1311356 10.1099/0022-1317-73-2-269

[18] Rixon FJ, Addison C, McLauchlan J (1992) Assembly of enveloped tegument structures (L particles) can occur independently of virion maturation in herpes simplex virus type 1-infected cells. J Gen Virol 73: 277–284. 1311357 10.1099/0022-1317-73-2-277

[19] Duckers DF, Meij P, Vervoort MBHJ, Vos W, Scheper RJ, et al (2000) Direct immunosuppressive effects of EBV-encoded latent membrane protein 1. J Immunol 165: 663–670. 10878338 10.4049/jimmunol.165.2.663

[20] Pegtel DM, Cosmopoulos K, Thorley-Lawson DA, van Eijndhoven MAJ, Hopmans ES, et al (2010) Functional delivery of viral miRNAs via exosomes. Proc Natl Acad Sci USA 107: 6328–6333. 20304794 10.1073/pnas.0914843107 PMC2851954

[21] Meckes DG, Raab-Traub N (2011) Microvesicles and viral infection. J Virol 85: 12844–12854. 21976651 10.1128/JVI.05853-11 PMC3233125

[22] Wurdinger T, Gatson NN, Balaj L, Kaur B, Breakefield XO, et al (2012) Extracellular vesicles and their convergence with viral pathways. Adv Virol 2012: 767694. 22888349 10.1155/2012/767694 PMC3410301

[23] Johnson R, Kaneene JB (1991) Bovine leukemia virus. Part 1. Descriptive epidemiology, clinical manifestations, and diagnostic tests. Compend Contin Educ Pract Vet 13: 315–327.

[24] Burny A, Cleuter Y, Kettmann R, Mammerickx M, Marbaix G, et al (1988) Bovine leukaemia: facts and hypotheses derived from the study of an infectious cancer. Vet Microbiol 17: 197–218. 2847391 10.1016/0378-1135(88)90066-1

[25] Kohara J, Konnai S, Onuma M (2006) Experimental transmission of bovine leukemia virus in cattle via rectal palpation. Jpn J Vet Res 54: 25–30. 16786975

[26] Lassauzet MLG, Thurmond MC, Johnson WO, Stevens F, Picanso JP (1990) Effect of brucellosis vaccination and dehorning on transmission of bovine leukemia virus in heifers on a California dairy. Can J Vet Res 54: 184–189. 2155048 PMC1255626

[27] Ferrer JF, Piper CE (1981) Role of colostrum and milk in the natural transmission of the bovine leukemia virus. Cancer Res 41: 4906–4909. 6272983

[28] Kuckleburg CJ, Chase CC, Nelson EA, Marras SAE, Dammen MA, et al (2003) Detection of bovine leukemia virus in blood and milk by nested and real-time polymerase chain reactions. J Vet Diagn Invest 15: 72–76. 12580302 10.1177/104063870301500117

[29] Meas S, Usui T, Ohashi K, Sugimoto C, Onuma M (2002) Vertical transmission of bovine leukemia virus and bovine immunodeficiency virus in dairy cattle herds. Vet Microbiol 84: 275–282. 11731179 10.1016/s0378-1135(01)00458-8

[30] Yamada T, Inoshima Y, Matsuda T, Ishiguro N (2012) Comparison of methods for isolating exosomes from bovine milk. J Vet Med Sci 74: 1523–1525. 22785357 10.1292/jvms.12-0032

[31] Itohara S, Mizuno Y (1984) Dimethyl sulfoxide enhances syncytium formation induced by bovine leukemia virus. Microbiol Immunol 28: 251–255. 6328232 10.1111/j.1348-0421.1984.tb00677.x

[32] Shimizu M, Satou K (1987) Frequency of persistent infection of cattle with bovine viral diarrhea-mucosal disease virus in epidemic areas. Jpn J Vet Sci 49: 1045–1051. 10.1292/jvms1939.49.1045 2828731

[33] Van Der Maaten MJ, Miller JM (1975) Replication of bovine leukemia virus in monolayer cell cultures. Bibl Haematol 43: 360–362. 10.1159/000399166 183699

[34] Aoki N, Kuroda H, Urabe M, Taniguchi Y, Adachi T, et al (1994) Production and characterization of monoclonal antibodies directed against bovine milk fat globule membrane (MFGM). Biochim Biophys Acta 1199: 87–95. 8280760 10.1016/0304-4165(94)90101-5

[35] Murakami K, Okada K, Ikawa Y, Aida Y (1994) Bovine leukemia virus induces CD5^−^ B cell lymphoma in sheep despite temporarily increasing CD5^+^ B cells in asymptomatic stage. Virology 202: 458–465. 7516599 10.1006/viro.1994.1362

[36] Mohan M, Malayer JR, Geisert RD, Morgan GL (2001) Expression of retinol-binding protein messenger RNA and retinoic acid receptors in preattachment bovine embryos. Mol Reprod Dev 60: 289–296. 11599039 10.1002/mrd.1090

[37] Hanaichi T, Sato T, Iwamoto T, Malavasi-Yamashiro J, Hoshino M, et al (1986) A stable lead by modification of Sato's method. J Electron Microsc 35: 304–306. 2440973

[38] Vermeire J, Naessens E, Vanderstraeten H, Landi A, Iannucci V, et al (2012) Quantification of reverse transcriptase activity by real-time PCR as a fast and accurate method for titration of HIV, lenti- and retroviral vectors. PLoS One 7: e50859. 23227216 10.1371/journal.pone.0050859 PMC3515444

[39] Pizzato M, Erlwein O, Bonsall D, Kaye S, Muir D, et al (2009) A one-step SYBR green I-based product-enhanced reverse transcriptase assay for the quantification of retroviruses in cell culture supernatants. J Virol Methods 156: 1–7. 19022294 10.1016/j.jviromet.2008.10.012

[40] Michishita K, Oohashi H, Minamisawa N, Katou R, Kimura N, et al (2011) Detection of bovine leukemia virus (BLV) DNA in milk derived from BLV-infected cows in a slaughterhouse. J Vet Med 64: 815–819 (in Japanese, with English summary).

[41] Kettmann R, Portetelle D, Mammerickx M, Cleuter Y, Dekegel D, et al (1976) Bovine leukemia virus: An exogenous RNA oncogenic virus. Proc Natl Acad Sci USA 73: 1014–1018. 57616 10.1073/pnas.73.4.1014 PMC430190

[42] Gould SJ, Booth AM, Hildreth JEK (2003) The Trojan exosome hypothesis. Proc Natl Acad Sci USA 100: 10592–10597. 12947040 10.1073/pnas.1831413100 PMC196848

[43] Nguyen DG, Booth A, Gould SJ, Hildreth JEK (2003) Evidence that HIV budding in primary macrophages occurs through the exosome release pathway. J Biol Chem 278: 52347–52354. 14561735 10.1074/jbc.M309009200

[44] Hanayama R, Tanaka M, Miwa K, Shinohara A, Iwamatsu A, et al (2002) Identification of a factor that links apoptotic cells to phagocytes. Nature 417: 182–187. 12000961 10.1038/417182a

[45] Reinhardt TA, Lippolis JD, Nonnecke BJ, Sacco RE (2012) Bovine milk exosome proteome. J Proteomics 75: 1486–1492. 22129587 10.1016/j.jprot.2011.11.017

[46] Hata T, Murakami K, Nakatani H, Yamamoto Y, Matsuda T, et al (2010) Isolation of bovine milk-derived microvesicles carrying mRNAs and microRNAs. Biochem Biophys Res Commun 396: 528–533. 20434431 10.1016/j.bbrc.2010.04.135

[47] Kosaka N, Izumi H, Sekine K, Ochiya T (2010) microRNA as a new immune-regulatory agent in breast milk. Silence 1: 7. 20226005 10.1186/1758-907X-1-7 PMC2847997

[48] Zhou Q, Li M, Wang X, Li Q, Wang T, et al (2012) Immune-related microRNAs are abundant in breast milk exosomes. Int J Biol Sci 8: 118–123. 22211110 10.7150/ijbs.8.118 PMC3248653

[49] Perez-Gonzalez R, Gauthier SA, Kumar A, Levy E (2012) The exosome secretory pathway transports amyloid precursor protein carboxyl-terminal fragments from the cell into the brain extracellular space. J Biol Chem 287: 43108–43115. 23129776 10.1074/jbc.M112.404467 PMC3522305

[50] Eldh M, Lötvall J, Malmhäll C, Ekström K (2012) Importance of RNA isolation methods for analysis of exosomal RNA: Evaluation of different methods. Mol Immunol 50: 278–286. 22424315 10.1016/j.molimm.2012.02.001

[51] Taylor DD, Zacharias W, Gercel-Taylor C (2011) Exosome isolation for proteomic analyses and RNA profiling. Methods Mol Biol 728: 235–246. 21468952 10.1007/978-1-61779-068-3_15

[52] Wiley RD, Gummuluru S (2006) Immature dendritic cell-derived exosomes can mediate HIV-1 trans infection. Proc Natl Acad Sci USA 103: 738–743. 16407131 10.1073/pnas.0507995103 PMC1334656

[53] Alais S, Simoes S, Baas D, Lehmann S, Raposo G, et al (2008) Mouse neuroblastoma cells release prion infectivity associated with exosomal vesicles. Biol Cell 100: 603–615. 18422484 10.1042/BC20080025

[54] Fevrier B, Vilette D, Archer F, Loew D, Faigle W, et al (2004) Cells release prions in association with exosomes. Proc Natl Acad Sci USA 101: 9683–9688. 15210972 10.1073/pnas.0308413101 PMC470735

