# Supplementary material for: Cell Infectivity in Relation to Bovine Leukemia Virus gp51 and p24 in Bovine Milk Exosomes
Source: PLoS One. 2013 Oct 17;8(10):e77359. doi: 10.1371/journal.pone.0077359 (PMC3798320; doi:10.1371/journal.pone.0077359)
Supplement: Table S1 — Primers used in this study. (DOC) [file pone.0077359.s001.doc]

Supporting Information

Table S1. Primers used in this study

Primers Target^a^  Sequence (5’ to 3’) Reference

BLV AF BLV pX CAG ACA CCA GGG GAG CCA TA 35

BLV BR CTG CTA GCA ACC AAT TCG GA

BLV CnF AGC CAT ACG TTA TCT CTC CA

BLV DnR CAG GTT AGC GTA GGG TCA TG

boGAPDH F GAPDH CCT TCA TTG ACC TTC ACT ACA TGG TCT A 36

boGAPDH R GCT GTA GCC AAA TTC ATT GTC GTT ACC A

MS2 forward MS2 TCC TGC TCA ACT TCC TGT CGA G 38

MS2 reverse CAC AGG TCA AAC CTC CTA GGA ATG

^a^ GAPDH, glycelaldehyde-3-phosphate dehydrogenase.
