# Supplementary figures and images for: Cell Infectivity in Relation to Bovine Leukemia Virus gp51 and p24 in Bovine Milk Exosomes
Source: PLoS One. 2013 Oct 17;8(10):e77359. doi: 10.1371/journal.pone.0077359 (PMC3798320; doi:10.1371/journal.pone.0077359)

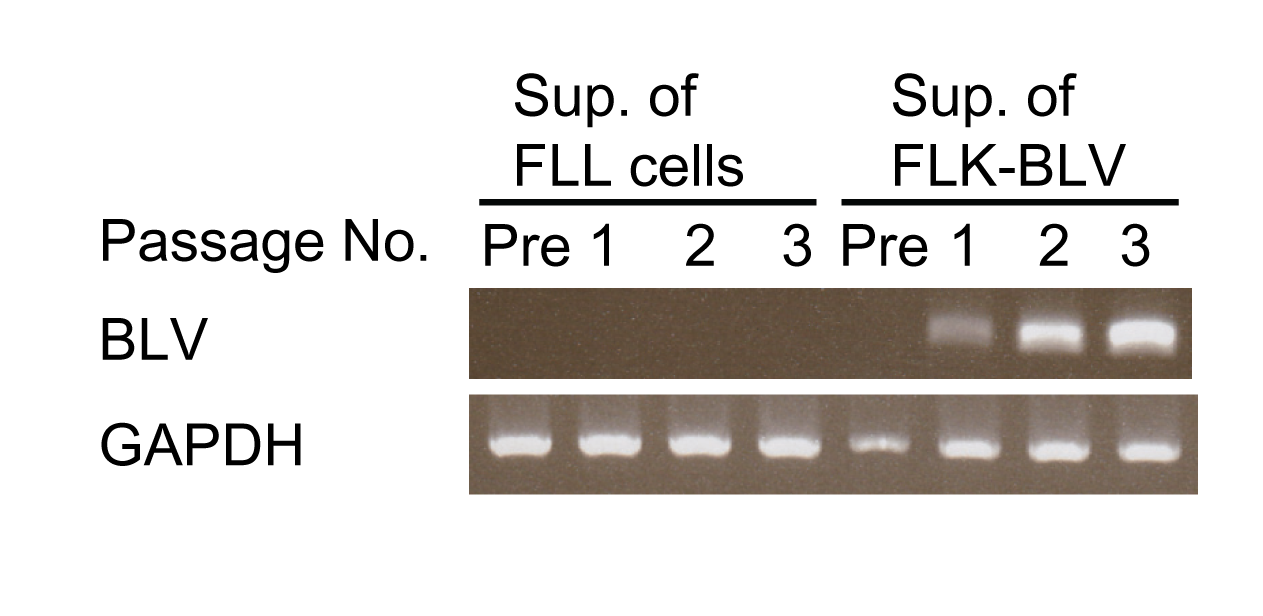

Supplement: Figure S1 — Infectivity assay, using FLL cells inoculated with the supernatant (sup.) of FLK-BLV cells. BLV DNA was detected in passaged FLL cells after inoculation of supernatant of FLK-BLV cells but not in cells inoculated with supernatant of FLL cells as a control by nested PCR targeting the pX gene. Detection of the GAPDH gene in DNA from FLL cells was used as an internal control for the PCR reaction. (TIF) [file pone.0077359.s002.tif]
